# An Investigation of Atomic Structures Derived from X-ray Crystallography and Cryo-Electron Microscopy Using Distal Blocks of Side-Chains

**DOI:** 10.3390/molecules23030610

**Published:** 2018-03-08

**Authors:** Lin Chen, Jing He, Salim Sazzed, Rayshawn Walker

**Affiliations:** 1 Department of Mathematics and Computer Science, Elizabeth City State University, Elizabeth City, NC 27909, USA; rrwalker135@students.ecsu.edu; 2 Department of Computer Science, Old Dominion University; Norfolk, VA 23529, USA; jhe@cs.odu.edu (J.H.); ssazz001@odu.edu (S.S.)

**Keywords:** protein, structure, cryo-electron microscopy, validation, statistics, X-ray, crystallography, side chain

## Abstract

Cryo-electron microscopy (cryo-EM) is a structure determination method for large molecular complexes. As more and more atomic structures are determined using this technique, it is becoming possible to perform statistical characterization of side-chain conformations. Two data sets were involved to characterize block lengths for each of the 18 types of amino acids. One set contains 9131 structures resolved using X-ray crystallography from density maps with better than or equal to 1.5 Å resolutions, and the other contains 237 protein structures derived from cryo-EM density maps with 2–4 Å resolutions. The results show that the normalized probability density function of block lengths is similar between the X-ray data set and the cryo-EM data set for most of the residue types, but differences were observed for ARG, GLU, ILE, LYS, PHE, TRP, and TYR for which conformations with certain shorter block lengths are more likely to be observed in the cryo-EM set with 2–4 Å resolutions.

## 1. Introduction

Cryo-electron microscopy (cryo-EM) is an emerging structure determination technique in addition to two other techniques: X-ray crystallography (X-ray) and Nuclear Magnetic Resonance (NMR). Currently, over 1900 atomic structures have been derived from electron density maps produced using cryo-EM technique, and they are deposited in Protein Data Bank (PDB) [1]. Some of the atomic structures are derived from cryo-EM density maps with 2–4 Å resolutions. Others are obtained from density maps of much lower resolutions. It is generally expected that atomic structures that are derived from high-quality density maps are more accurate than those derived from lower-quality maps. Although there are more than 5700 EM density maps in the EM Data Bank (EMDB) as of January 2018, about 54% of them have resolution lower than 10 Å and 24% with 5–10 Å resolution [2]. The set of highest-quality cryo-EM density maps contains those with 2–4 Å resolutions. The number of such high-quality maps has increased rapidly since 2012. As of December 2016 when the data were downloaded for the study, there were 416 cryo-EM density maps with better than or equal to 4 Å resolutions. Some of them are protein structures and others are nucleic acid structures. Atomic structures derived from these maps represent a set of most accurate structures derived from cryo-EM density maps.

Although both X-ray crystallography and cryo-EM produce electron density maps from which atomic structures are derived, they have different sample preparation, different data collection, and different data processing. In addition, structure changes have been observed at different temperatures [3]. The concept of resolution in X-ray depends on the highest resolvable diffraction spots. However, a commonly used method to evaluate resolution for a cryo-EM density map, Fourier Shell Correlation [4], quantifies the correlation of two halves of the data set in Fourier space. Regardless of the differences between the two structure determination methods, side chains are generally distinguishable in a cryo-EM density map with about 3 Å resolution [5,6,7,8,9]. It is an open question how accurate cryo-EM structures are, particularly for those derived from the highest-quality cryo-EM density maps.

Validation of protein structures is an important step to maintain an accurate archive of atomic structures [10,11,12,13,14]. Various analysis tools have been developed since 1990 to identify outliers [12,15,16,17,18,19,20,21,22,23,24,25]. In order to create comprehensive assessment criteria, Worldwide Protein Data Bank (wwPDB) convened a Validation Task Force (VTF) to establish policies, standards, and specifications of formats. The VTF includes X-ray VTF, NMR VTF, and 3DEM VTF. In 2011, X-ray VTF published its recommendation report on how to validate protein structures derived from X-ray density maps [26]. After that, 3DEM VTF and NMR VTF released their recommendation reports in 2012 [27] and 2013 [28]. The wwPDB accepts and curates depositions using wwPDB Deposition & Annotation (D&A) system that implements the recommendations from wwPDB VTF [29]. wwPDB generates validation reports for new depositions containing the results from rigorous tests of structure model quality. The validation reports for X-ray structures have been updated with the D&A system in March 2017 with 2016 statistics. The validation reports for NMR and 3DEM structures in PDB were available since May 2016.

3DEM VTF recommended to validate both EM density map and models. Several methods have been proposed to prevent overfitting of data: cross-validation [27], limit of the reconstruction with low signal-to-noise-ratios [30], and regulation using a Gaussian [31]. Practices implemented to validate density maps include absolute hand determination [32,33], data coverage and agreement between images and class averages [34], and statistical assessment of maps [35]. Rfree [36] factor is used to check the correlation coefficient between a model and its cryo-EM map. According to wwPDB EM validation reports, EM models are often validated using existing tools for X-ray data and the correlation coefficient between an EM density map and its atomic model [37]. 

As more atomic structures are determined using high-quality cryo-EM density maps, it is becoming possible to characterize statistical behaviors of such structures. In this paper, we present a statistical analysis of structures derived from ultra-high-resolution density maps of X-ray and those derived from EM density maps with 2–4 Å resolutions. The analysis uses the length of the distal block of a side-chain in addition to backbone and side-chain torsion angles to characterize the conformational distribution of each amino acid.

## 2. Results

Two X-ray data sets and two EM data sets were analyzed in this study. An X-ray data set, referred as X-ray-1.5, contains 9131 PDB protein structures that are solved using X-ray crystallography and have a resolution better than or equal to 1.5 Å. The protein structures were extracted from the PDB website using the default sequence similarity value of 90%. X-ray-1.5 was used as the reference in the study because side-chain positions are more precisely defined at such resolutions than lower resolutions. In order to characterize the statistics from structures that are derived from high quality EM density maps, we downloaded an EM data set in December 2016, referred as EM-2-4, containing 237 PDB protein structures that are derived from EM density maps with resolutions between 2 and 4 Å (including 4 Å). These maps represent the most accurate structures derived from cryo-EM density maps. The third data set, referred as EM-4-6, contains 168 protein structures derived from EM density maps with resolutions greater than 4 Å and less than or equal to 6 Å resolutions. The fourth data set, referred as X-ray-237, contains 237 protein structures that are randomly picked from the 9131 X-ray structures that are used in the first data set.

### 2.1. 3D Scatter Plots for Backbone and Side-Chain Torsion Angles

The conformation of a residues is widely used to validate the quality of a model. One of the practices in the validation pipeline uses combination of torsion angles from either the backbone or the side-chain. Most of modeling methods have considered Ramachandran criteria [38] and side-chain rotamer library [39] to assign favorable combination values to residues.

**Figure 1:**
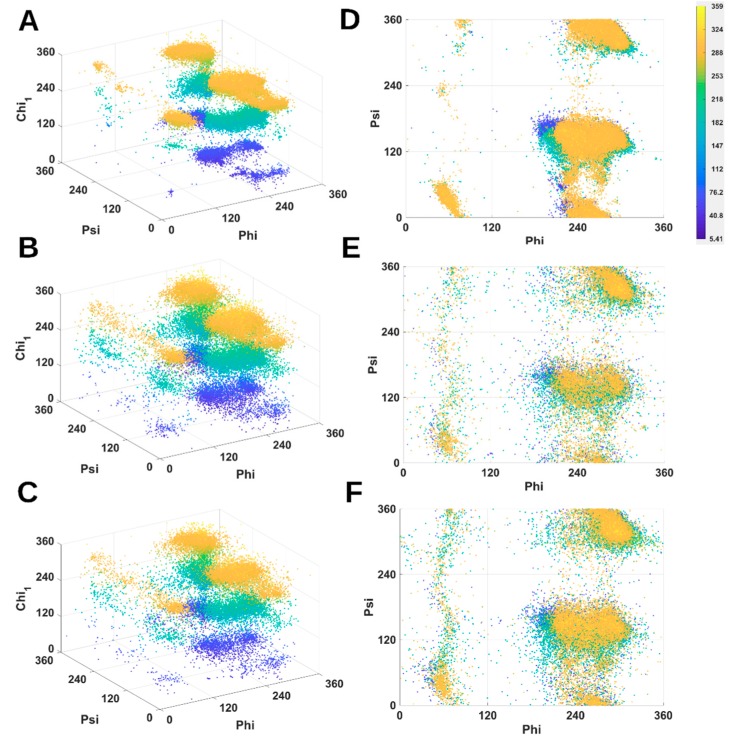
3D scatter plots and Ramachandran plots of Arginine (ARG) residue for three data sets. 3D scatter plots of (φ, ψ, ***χ*_1_**) are shown for X-ray-1.5 data set (**A**); EM-2-4 data set (**B**); and EM-4-6 data set (**C**); Ramachandran plots of (φ, ψ) are shown for X-ray-1.5 data set (**D**); EM-2-4 data set (**E**); and EM-4-6 data set (**F**). The color code (shown) is based on $\chi_{1}$, and it is the same for each panel.

We investigated the distribution of combined torsion angles (φ, ψ, $\chi_{1}$) of arginine (ARG) using a 3D scatter plot (Figure 1). Each conformation of ARG is represented as a point in the plot. For clear visualization, points are colored according to $\chi_{1}$ angle. Although the X-ray-1.5 data set contains the most number of proteins as compared to EM-2.4 and EM-4-6 data sets, the points are highly clustered within several areas. ARG conformations are rarely observed outside those areas (Figure 1A). Due to EM data set has much lower resolution, EM points are less concentrated in those clusters and more conformations are seen in between clusters as expected (Figure 1B,C). As an example for the top layer (yellow), there are two large clusters at (250°, 330°, 300°), (250°, 150°, 300°), one small cluster at (50°, 50°, 300°), and two weak clusters at (80°, 350°, 300°) and (50°, 230°, 300°). In the same layer for the two EM data sets (Figure 1B,C), there is a belt spanning from about (50°, 0°, 300°) to (50°, 360°, 300°). The two clusters at (250°, 330°, 300°) and (250°, 150°, 300°) are broader (Figure 1B,C) suggesting that many ARG conformations are unfavorable according to the distribution of X-ray data. The middle layer (cyan in Figure 1A) contains a cluster at (290°, 330°, 180°) and a larger cluster spanning from about (200°, 140°, 180°) to (300°, 140°, 180°). The middle layer of EM data (Figure 1B,C) has the cluster spanning from (200°, 330°, 180°) to (330°, 330°, 180°) and a large cluster spanning (200°, 140°, 180°) to (320°, 140°, 180°). EM data show a “cyan belt” in the middle layer from (50°, 0°, 180°) to (50°, 360°, 180°) (Figure 1B) which does not exist in the corresponding space in Figure 1A. In the bottom layer (blue), X-ray points have three clusters at about (300°, 330°, 50°), (200°, 150°, 50°) and (300°, 150°, 50°). EM points show broader blue clusters compared to those of X-ray data.

Although current modeling and validation methods have considered favorable residue conformations using Ramachandran criteria and rotamer libraries, more combinations of torsion angles should be considered as more data are available. Using the Ramachandran plot that is colored based on $\chi_{1}$, we observed that the X-ray data set and the EM data sets generally have a similar distribution of (φ, ψ) (Figure 1D–F). However, EM-2-4 set has many points outside the clusters of X-ray-1.5 set. The broader clusters of EM data sets shown in the 3D scatter plot (Figure 1A–C) and the colored Ramachandran plot (Figure 1D–F) suggest that some conformations of ARG are unfavorable if X-ray high-resolution data are used as a reference. EM structures may benefit from a refinement of side-chain conformations if more dependency of torsion angles is incorporated in model building.

### 2.2. Normalized Probability Density Function for Block Lengths

**Figure 2:**
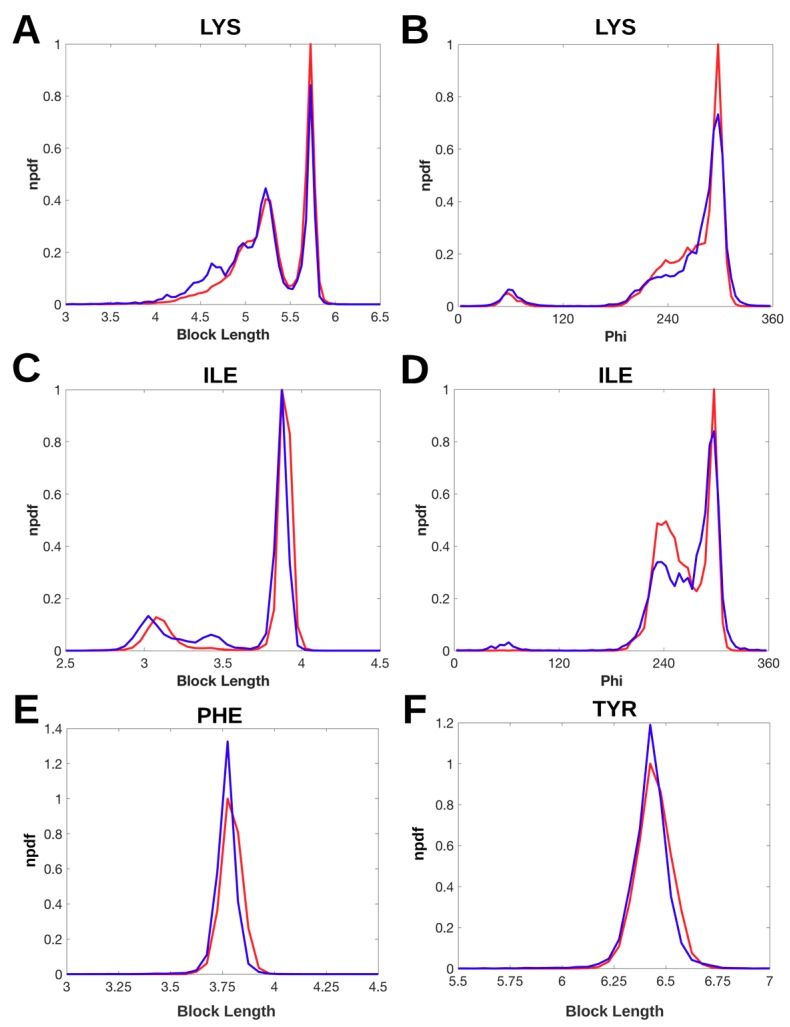
Normalized probability density function of block length and torsion angle φ. The distribution of block length and φ is shown respectively as a probability density function (npdf) normalized by the highest peak of the pdf of the X-ray-1.5 data set. The npdf of X-ray-1.5 data set (red line) and the npdf of EM-2-4 data set (blue line) are shown for lysine (LYS) (**A**,**B**); isoleucine (ILE) (**C**,**D**); phenylalanine (PHE) (**E**); and tyrosine (TYR) (**F**) respectively.

We calculated block length ($d_{\mathrm{Block}}$) using the distal block of a side-chain for each of 18 types of residues (see Materials and Methods), since the position and orientation of the distal block are sensitive in distinguishing side-chain conformations [40]. The block lengths of lysine (LYS) range from 4 Å to 6 Å approximately. The side-chains of LYS show two peaks at 5.2 Å and 5.7 Å respectively in the normalized probability density function (npdf) from both the EM-2-4 data set and the X-ray-1.5 data set, suggesting two preferred block lengths (Figure 2A). The height of the peak at 5.7 Å is lower in the npdf of the EM-2-4 set than that of the X-ray-1.5 set. This suggests that it is less likely to find a conformation of the side-chain with block length of 5.7 Å in the EM-2-4 data set than in the X-ray-1.5 set. However, the difference in the height of the highest peak only reflects the situation at one point (the peak). When Figure 2A and 2B are both considered that the most preferred conformation of LYS has (φ, $d_{\mathrm{Block}}$) as (298°, 5.7 Å). The EM-2-4 set has lower probability of having the most preferred conformation than the X-ray-1.5 set. In the region where $4\;Å<d_{\mathrm{Block}}\leq 5.2\;Å$, the EM curve appears to be mostly above the X-ray curve (Figure 2A and Figure S1) indicating that most points in this region (that corresponds to shorter block lengths) show higher probability in the EM-2-4 set than in the X-ray-1.5 and X-ray-237 sets. In the range between 4 Å and 4.8 Å, npdf value of X-ray-1.5 is smaller than that of npdf of EM-2-4 (Figure 2A). This suggests that it is more likely to observe a shorter side-chain for LYS, within the range of 4 Å to 4.8 Å, in the EM-2-4 set than in the X-ray-1.5 set. This may suggest that the visibility of the density map at the side chain region may have given preference to a shorter LYS side-chain when modeling some EM structures.

The block length of isoleucine (ILE) is about 2.8 Å-4 Å (Figure 2C). The height of the peak at 3.88 Å of block length is about the same for both X-ray-1.5 set and the EM-2-4 set. The right side of the curve falls faster in the EM-2-4 set (blue) than in the X-ray-1.5 set (red) (Figure 2C). This suggests that it is less likely to observe those side-chain conformations with block length between 3.88 and 4 Å in the EM-2-4 data set. There is a small peak at about 3.4 Å block length for EM structures that is not obvious for X-ray structures. The peak of the X-ray npdf at 3.08 Å of block length shifts slightly left towards shorter block lengths. This suggests that it is more likely to assign a side-chain conformation of ILE with a slightly shorter block length at about 3 Å in the EM-2-4 data set. The npdf of backbone φ angle also shows some differences between the X-ray structures and EM structures for ILE, such as at 62.5° of φ angle, the unfavorable angle if judged by the X-ray curve (red) (Figure 2D).

**Figure 3:**
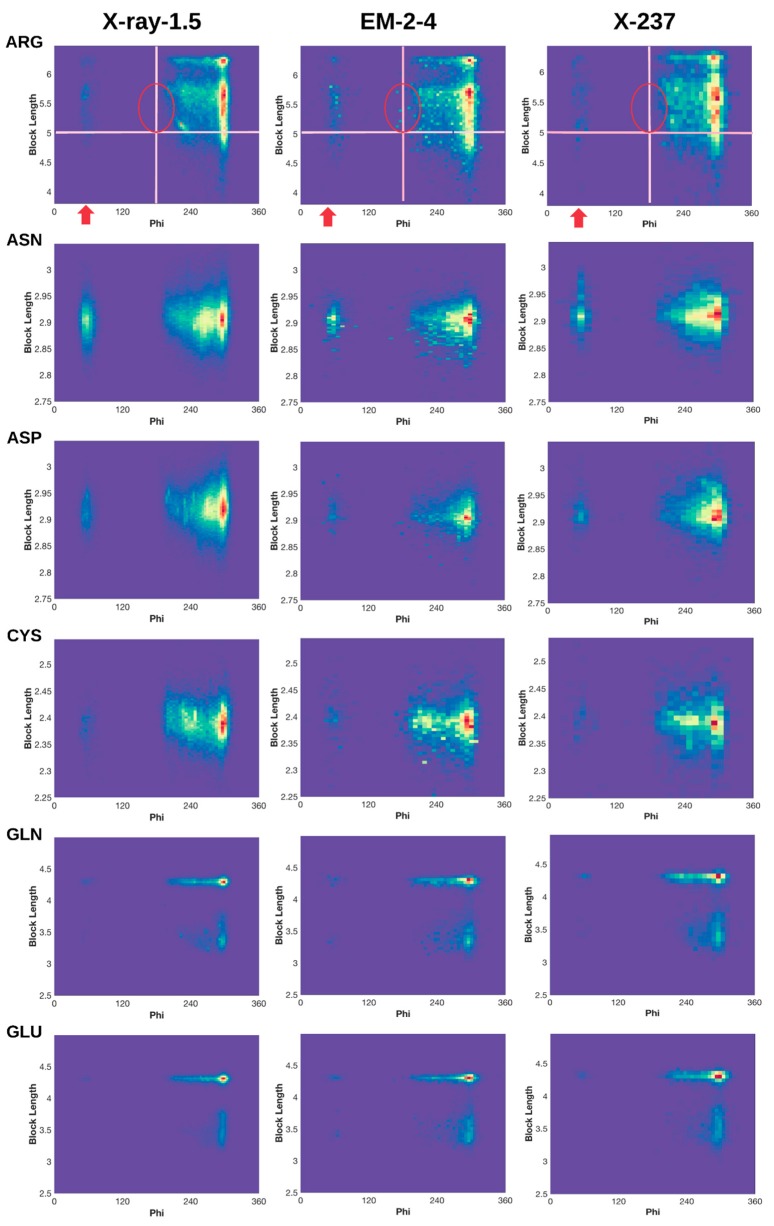

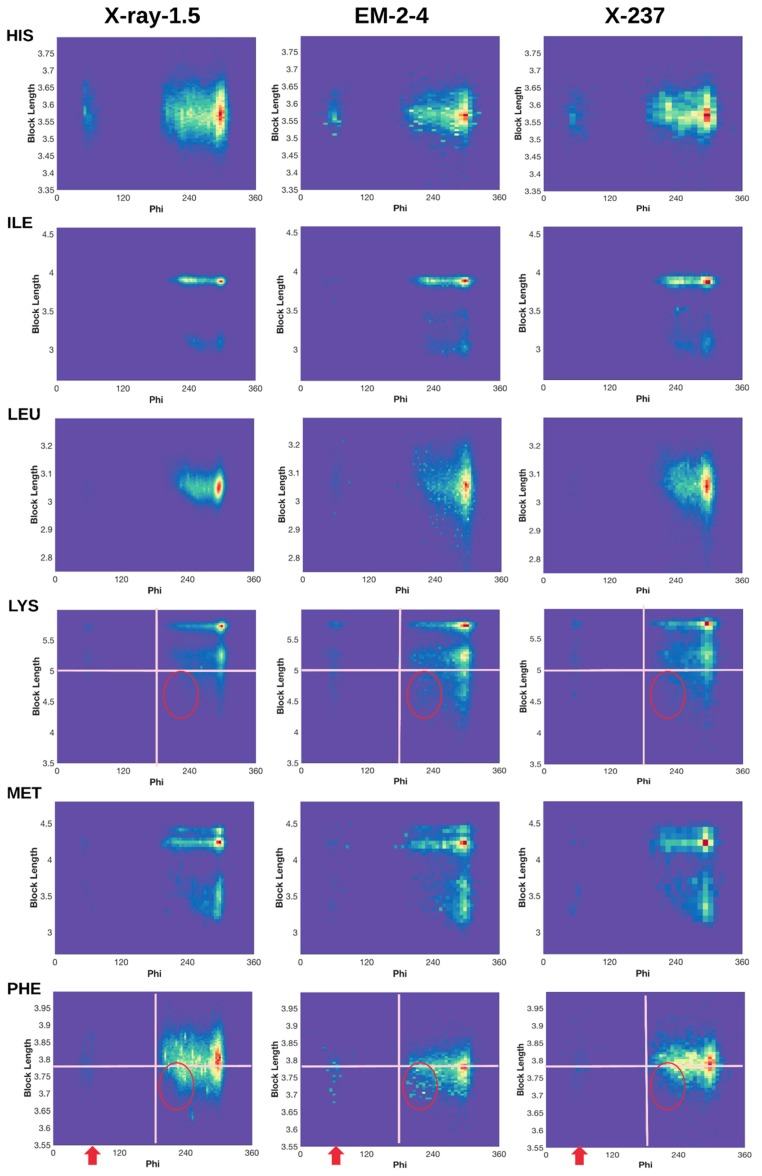

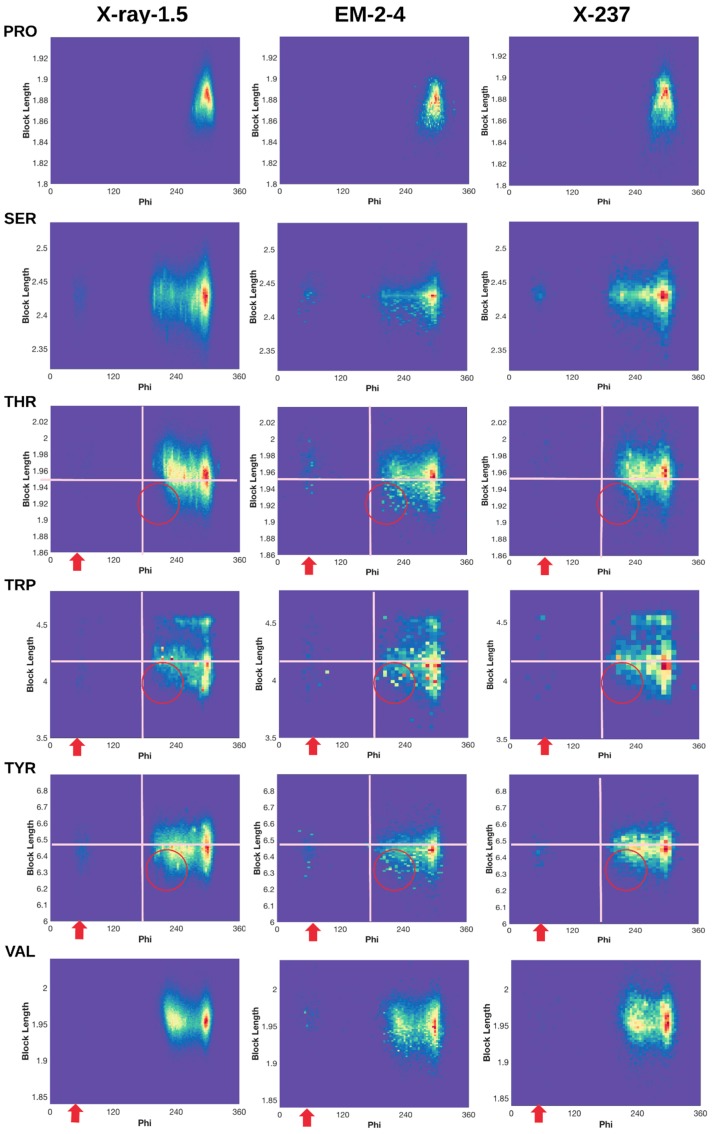
Normalized 2D histogram of (**ϕ**, **d_Block_**) for 18 residue types (Alanine (ALA) and Glycine (GLY) not included). Normalized 2D histograms of X-ray-1.5 data set (left column), EM-2-4 data set (middle column), and X-ray-237 data set (right column) were plotted using the ndhist function of MATLAB such that the most popular (**ϕ**, **d_Block_**) is colored red and the least popular is in dark blue [41]. Visually observed difference regions are labeled with red arrows and circles. Cross lines are drawn to assist comparison.

The npdf of block length shows that both X-ray-1.5 set and the EM-2-4 set have the same peaks located at 3.78 Å for phenylalanine (PHE) and 6.43 Å for Tyrosine (TYR) respectively (Figure 3E,F). However, the EM-2-4 data set has a higher peak than the X-ray-1.5 set for both PHE and TYR. This suggests that it is more likely to assign a side-chain with the most popular conformation in the EM structures than in the X-ray structures. The curves at the right side of the peak fall faster in the EM-2-4 set than in the X-ray-1.5 set. This suggests that the probability of finding side-chain with a block length longer than the most popular length is smaller in the EM-2-4 set than in the X-ray-1.5 set.

In general, the lower the resolution of the density map, the more challenging to determine precisely the position of the distal block in electron density maps. The lengths of distal blocks were characterized from X-ray-1.5 set containing 9131 proteins and EM-2-4 set containing 237 proteins. We observed that the EM-2-4 set has bias towards those side-chain conformations with slightly shorter block lengths for LYS, ILE, PHE, and TYR. For example in the case of LYS, EM-2-4 has lower probability at 5.7 Å block length but higher probability at 4-5.2 Å when it is compared to X-ray-1.5 set. It is possible that some side-chain conformations with long block length of about 5.7 Å were modeled to shorter side-chains. The bias is less likely due to the difference in the number of samples for the two data sets (see Supplementary Figure S1). We randomly selected 237 proteins from the X-ray-1.5 set to create X-ray-237 set that is expected to have the same magnitude of residues as for the EM-2-4 set. Yet the same bias towards shorter block length was observed when the X-ray-237 and EM-2-4 were compared (Supplementary Figure S1). In addition to those four residues, we also observed higher chance of finding shorter side-chain for ARG, Glutamic acid (GLU) and Tryptophan (TRP) (see Supplementary Figure S3). No obvious difference was observed in the length distributions of the other 11 residues (see Supplementary Figure S2).

### 2.3. 2D Histograms of Combined Features Using the Block Length and Backbone Torsion φ

We characterized (φ,$\;d_{\mathrm{Block}}$) profile for each of the 18 residues (Figure 3), since the 2D histogram reflects the dependency between the backbone torsion angle φ and the side-chain conformation. The two residues that are not characterized for block length are the two small residues GLY and ALA. A normalized 2D histogram was obtained by normalizing using the value of the highest peak (shown in red). Therefore, the color at each point of the 2D histogram represents a population ratio with respect to the population of the most popular conformation. The 2D histogram of (φ, $d_{\mathrm{Block}}$) demonstrates a characteristic pattern for each residue. For example, the pattern of ARG is quite different from that of ASN (Figure 3 row 1 and row 2), suggesting that the 2D histogram captures the distribution of conformations despite of its simplicity.

#### 2.3.1. Conformations of Side-Chains When φ is Near 60°

Although the backbone torsion angle φ is mostly negative in the classical view of Ramachandran plot in which φ is between −180° to 180°, positive φ exist and they mostly cluster around 60°. The eighteen 2D histograms derived from 9131 ultra-high-resolution structures show that the population of φ near 60° exists but it is extremely low, as seen from the dark blue or blue color for all eighteen residues except ASN, ASP, and HIS (Figure 3 left column). In the case of ARG, those side-chain conformations are highly restricted in specific clusters when φ is about 60° (Figure 1A). The low population of such conformations may present a challenge in assignment of such conformations during structural determination. Higher population ratio of φ was observed in the EM-2-4 data set for ARG, PHE, THR, TRP, and TYR when φ is about 60°. As an example for ARG, there is a more obvious “vertical belt” at about 60° in the histogram of EM-2-4 (middle panel of row 1) than that in the histogram of X-ray-1.5 (left panel of row 1) (Figure 3). In fact, a few spots in the “vertical belt” appear yellow indicating higher population ratio than those points in blue. The visual difference at this region is less likely due to the difference in total number of proteins included in the two data sets, since the “vertical belt” is not as obvious in the 2D histogram of X-ray-237 set that has the same number of proteins as in the EM-2-4 set. The “vertical belt” observed in the 2D histogram aligns with the observation in the 3D scatter plot of ARG (Figure 1B) in which more points appear to scatter at the region of φ near 60°.

Normalized 2D histogram of PHE shows that the population ratio at φ ~ 60° is higher in the EM-2-4 set than in the X-ray-1.5 or the X-ray-237 set. We observe a “belt” at the region where φ is between 40° and 70° in the EM-2-4 set (Figure 3). In fact, some of the points in the belt are light yellow. The most popular point (φ, $d_{\mathrm{Block}}$) in the “belt region” of EM-2-4 set is (58.04°, 3.79Å), and the most popular point of PHE is (301.8°, 3.78 Å). The population ratio between the two points is 18%, meaning the most popular configuration in the belt is 18% of the population of the most popular configuration of PHE. For X-ray-1.5 set, the most popular point in the belt is at (69.28°, 3.86 Å), and the most popular point (red peak in the left column of PHE row in Figure 3) is at (303.5°, 3.81 Å). The population ratio is 4.1%, more than 1/4 times lower than that of the EM-2-4 set.

#### 2.3.2. Shorter Side-Chains When φ is Near 210°

Each residue has a range of block lengths representing the overall lengths of different conformations. As an example, LYS has a block length ranging from about 4 Å to 5.8 Å (row LYS of Figure 3). When comparing the normalized 2D histograms of three data sets (the X-ray-1.5, EM-2-4, and X-237) four residues LYS, PHE, TRP, and TYR, show a higher population ratio at the region of φ near 210° for the shorter block lengths. For example, the population ratio of LYS is very low for those conformations with block length between 4 and 5 Å and φ between 200° and 230°, according to the X-ray-1.5 data set (the left column of row LYS in Figure 3). However, the population ratio is higher for about eight spots (in the circle) when the 2D histogram of EM-2-4 is compared with that of the X-ray-1.5 and X-ray-237. Since the ratio is calculated between the population at a point and the population at the peak, a higher ratio suggests either there is higher population in this region or the population is low at the peak. In this case, the block length at the peak is 5.8 Å that corresponds to an extended conformation of LYS. 

Similar observations are with PHE, TRP, and TYR when the residue has shorter block lengths and when φ is near 210°. For example, TYR block lengths range from about 6.2 Å to 6.8 Å. The population ratio at about (210°, 6.3 Å) is quite small according to X-ray-1.5 data set (left column of TYR row in Figure 3). However, the population ratio is higher at this region for the EM-2-4 data set. Our investigation suggests that one needs to be cautious when assigning a conformation with a short block length of the residue with backbone φ near 210°, since most of the residues have low probability of adopting such conformations.

## 4. Materials and Methods

**Figure 4:**
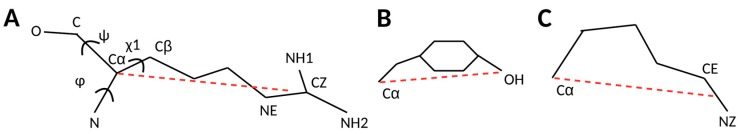
The four features—φ, ψ, ***χ*_1_**, and block length. The block length (dashed line) was calculated using the center of the distal block of the side chain, illustrated for ARG (**A**), TYR (**B**) and LYS (**C**).

The statistical analysis was implemented using protein structures downloaded from RCSB (www.rcsb.org). Four features, backbone torsion angle Phi φ and Psi ψ, side-chain torsion angle $\chi_{1}$, and block length $d_{\mathrm{Block}}$ were used in characterization of backbone-dependent side-chain conformations (Figure 4). Note that the range of φ in this paper is 0°–360°, instead of typical range of −180°–180° in the Ramachandran plot. In fact, $\varphi =\varphi_{R}$ when φ∈[0,180°], and $\varphi =\varphi_{R}+360{}^{\circ}\;$ when φ∈(180°, 360°), where φ and $\varphi_{R}$ are the torsion angle Phi in this paper and in the Ramachandran plot respectively.

A side-chain is divided into blocks and the position of the distal block was used to represent the conformation of a side-chain for 18 of the 20 residues [40]. GLY and ALA are not included due to the small size of their side-chains. The position and orientation of the distal block have been used previously to characterize conformations of side-chains in an energy function [40]. A side-chain block length is the distance between Cα and the mass center of the distal block of a residue (Figure 4).

Four data sets (X-ray-1.5, EM-2-4, EM-4-6 and X-ray-237) were created (see Results). For those structures solved using X-ray crystallography, the first chain of each protein was used and the first conformation of each side-chain was used if there are alternative conformations. Since the X-ray-1.5 set contains 9131 protein structures, the number of residues should be sufficient for statistical analysis. EM-2-4 set contains 237 protein structures. In order to include as many conformations as possible, we used all chains in each PDB file in the study. Although some chains are related by non-crystallographic symmetry and are expected to have overall similar structures, structural difference has been observed between different chains that are related by non-crystallographic-symmetry (NCS) [42]. However, further characterization with more EM structures is needed as they are becoming available.

For each of the four features, a probability density function (pdf) was generated for each of the 18 residues using X-ray-1.5 and EM-2-4 data sets respectively. A pdf was generated using a bin size of 5° for φ, ψ and $\chi_{1}$ and 0.05 Å for $d_{\mathrm{Block}}$. Normalized probability density functions (npdf) were derived using two probability density functions, one from the X-ray-1.5 set, and the other from the EM-2-4 set. Normalized pdf was calculated by dividing each pdf with the value of the highest peak of the X-ray-1.5 pdf. A total of 72 npdfs were generated for 18 types of residues and 4 features for each type. The python scripts and MATLAB scripts in the study have been deposited to Github at https://github.com/lin-chen-VA/structures. The plots and histograms will be maintained at http://www.cs.odu.edu/~jhe. The 2D histograms were plotted for each pair of features (φ, $\chi_{1}$), (ψ, $\chi_{1}$), (φ, ψ), (φ, $d_{\mathrm{Block}}$) and (ψ, $d_{\mathrm{Block}}$) using MATLAB ndhist function [41].

## 5. Conclusions

Precise characterization of the conformation of a residue involves the use of both backbone and side-chain torsion angles. Although there are sufficient (9131) X-ray structures in the X-ray-1.5 data set for statistical characterization, there are only 237 protein structures in the EM-2-4 data set. In order to sample conformations with sufficient statistics, we used the length of the distal block to represent the overall side-chain conformation. Although not as precise as using all torsion angles of the side-chain, it is a simple parameter to distinguish between a folded and an extended conformation of the side-chain. We characterized side-chain conformations using the length of the distal block for 18 of the 20 amino acids, in addition to torsion angle φ, ψ, and $\chi_{1}$ for the side-chain and backbone respectively.

The distribution of block lengths shows that the npdf of block lengths is similar between the X-ray-1.5 set and EM-2-4 set for most of the 18 residues. However, differences were observed for seven residue types – ARG, GLU, ILE, LYS, PHE, TRP, and TYR. The most popular block length is 5.7 Å and 3.88 Å for LYS an ILE respectively. Our results suggest that it is more likely to observe those conformations with block lengths between 4 Å to 5.2 Å for LYS and between 2.9 Å to 3.5 Å for ILE in the EM-2-4 set than in the X-ray-1.5 set. The most popular block length for PHE and TYR is 3.78 Å and 6.43 Å respectively. Our results show that it is less likely to observe those PHE and TYR conformations with block lengths longer than the most popular lengths in EM-2-4 set than in X-ray-1.5 set. Although it is generally challenging to assign side-chains accurately from a density map beyond 2 Å in resolution for both X-ray and cryo-EM density maps, our analysis suggests that those seven residues show statistical bias towards certain shorter side-chains. It is not clear if the bias toward shorter chain is related to the fitting process in which the density closer to the backbone may have dominated the assignment. Further investigation is needed for more accurate assignment of side-chains. 

Due to limited data available, the analysis of EM-2-4 set is based on 237 protein structures with all chains available in the PDB files. Ideally, those chains that are NCS-related should not be included, although they are often not identical. The first alternative is included if multiple alternatives are available. Though side-chain polymorphism [43] is common in models from high-resolution X-ray maps, alternative side-chain conformations are not prevalent in models from EM maps. Wlodawer et al. [44] reported differences between selected high-resolution density maps produced from X-ray crystallography and cryo-EM. This paper aims to characterize individual residues from a statistical perspective.

The number of atomic structures solved using cryo-EM technique has grown rapidly. Although structural validation guidelines are recommended for the newly solved cryo-EM structures, it is important to understand any statistical difference between the cryo-EM structures and most accurately determined X-ray structures. The ultra-high-resolution data of X-ray was used as a reference in this study. We report the systematic bias towards shorter side-chains in details for seven residues. The finding in this paper should be further verified when more cryo-EM structures are available. 

## Supplementary Materials

The following are available online.
