# Supplementary material for: An Investigation of Atomic Structures Derived from X-ray Crystallography and Cryo-Electron Microscopy Using Distal Blocks of Side-Chains
Source: Molecules. 2018 Mar 8;23(3):610. doi: 10.3390/molecules23030610 (PMC5967250; doi:10.3390/molecules23030610)
Supplement: Supplementary file 1 [file molecules-23-00610-s001.pdf]

Supplementary Materials

<u>Characterization of block lengths using X-ray-237 set for LYS, ILE, PHE, and TYR</u>

The sizes of two data sets in Figure 2 have different magnitudes. X-ray-1.5 has 9131 proteins and EM-2-4 has 237 proteins. Though two data sets are significant different in size, it is possible that 237 protein structures are sufficient to show meaningful statistical characters. To explore about the effect of data size, X-ray-237 was created by randomly sampling 237 proteins from the X-ray-1.5 set. EM-2-4 set and X-ray-237 set have the same number of proteins involved. The normalized probability density functions (npdfs) of block length in EM-2-4 and X-ray-237 are shown in Figure S1.

The two peaks of block length of lysine (LYS) at 5.2 Å and 5.7 Å in Figure 2A are observed at the same locations in Figure S1-LYS. We observe in both Figure 2A and Figure S1 that the npdf value of EM-2-4 set is higher than that of X-ray-1.5 (Figure 2A) and X-ray-237 (Figure 1S-LYS) when the block length is between 4 Å and 4.8 Å and at about 5.2 Å. The npdf of EM-2-4 has lower value than that of X-ray-237 at 5.5 Å (Figure S1-LYS). At this position, the npdf of EM-2-4 has the almost same value as that of X-ray-1.5 (Figure 2A). The difference of npdf value is caused by the random selected samples of X-ray-237. Similarly, the slight left shifts of peaks in EM-2-4 comparing with X-ray-1.5 in Figure 2 are also observed in Figure S1 for ILE, PHE, and TYR comparing with X-ray-237 respectively.

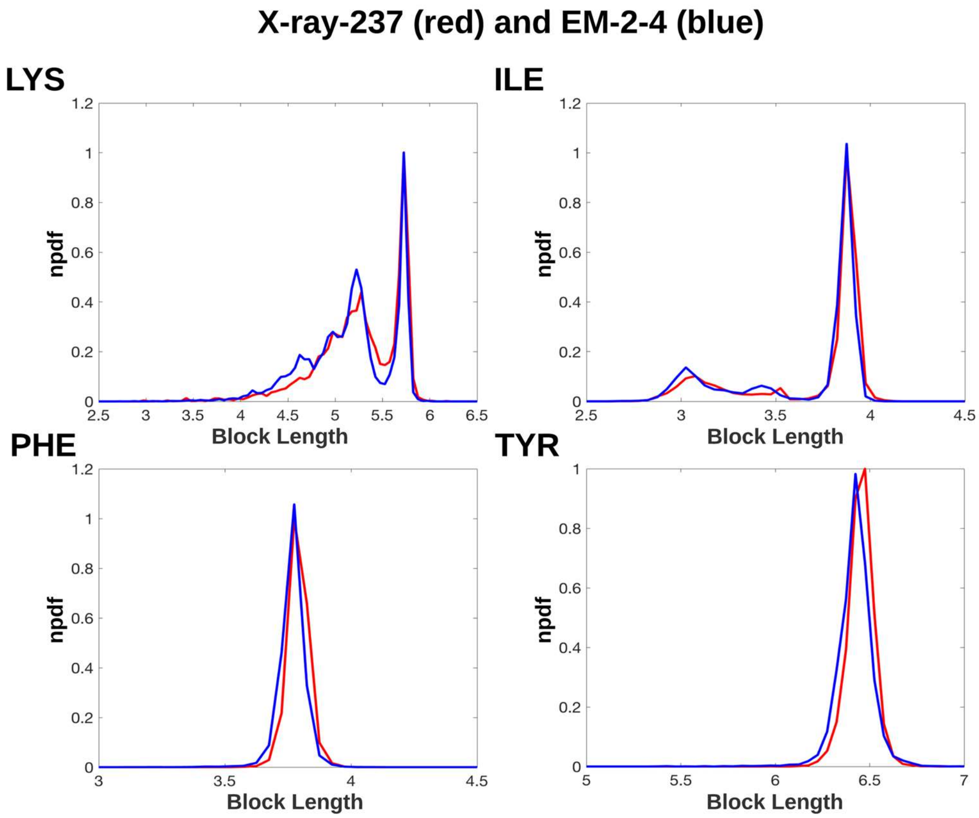

Figure S1. The npdf of X-ray-237 data set (red line) and the npdf of EM-2-4 data set (blue line) are shown for lysine (LYS), isoleucine (ILE), phenylalanine (PHE), and tyrosine (TYR) respectively.

Analysis of both X-ray-1.5 (9131 structures) and X-ray-237 (237 structures) shows that EM-2-4 set has similar bias towards those side-chain conformations with shorter block lengths when both Figure 2A and Figure S1 are considered.

Similar block length distributions for ASP, GLN, HIS, and MET

Although differences were observed for seven residues between the npdf of X-ray-1.5 and the npdf of EM-2-4, we did not observe obvious difference for the rest 11 residues (not include ALA and GLY). Figure S2 shows the block length npdf for four such examples. Aspartic acid (ASP) in both X-ray-1.5 and EM-2-4 has a single peak of npdf at 2.9 Å and similar peak shape. Similar distribution is observed for X-ray-237 set (red) and EM-2-4 set (blue) (Figure 2S row 1). Glutamine (GLN) has two peaks at 3.4 Å and 4.3 Å (Figure S2 row 2). Its npdfs of X-ray-1.5 and EM-2-4 (left column) have the same peak shape, though the peak of X-ray-1.5 has higher value than that of EM-2-4 at 4.3 Å. Similar shape of the curve was also observed in the plot for X-ray-237 set and EM-2-4 set (Figure 2S row 2 of right column). The similar block length npdfs in the left column and right column suggest that 237 proteins, although much less than 9131 proteins, captures the statistical characters of block lengths. No obvious difference has been observed in the length distributions of histidine (HIS) and methionine (MET) (Figure S2 row 3, 4).

Block length distributions for ARG, GLU, and TRP

The npdfs of arginine (ARG), glutamate (GLU), and tryptophan (TRP) for three data sets (X-ray-1.5, X-ray237, and EM-2-4) are shown in Figure S3. For ARG, the npdf of EM-2-4 has higher value, at 4.2 Å to 5.0 Å, than the npdfs of X-ray-1.5 (left column) and X-ray-237 (right column). This suggests that ARG in EM-2-4 is more likely to find a conformation within this range than X-ray-1.5 and X-ray-237. GLU has two unobvious peaks from 3.0 Å to 3.8 Å in the npdfs. In this area, the npdf of EM-2-4 has higher value than the npdfs of X-ray-1.5 and X-ray-237. The left shift is observed in the curve of npdf of EM-2-4 compared with the npdf curve of X-ray-1.5. This may suggest that it is more likely to have a shorter side-chain for GLU. For TRP, the two peaks are at 4.125 Å and 4.475 Å of the npdf of EM-2-4 corresponding to the two peaks located at 4.175 Å and 4.525 Å of the npdfs of X-ray-1.5 and X-ray-237. It also suggests that TRP has a preference of shorter side-chain when modeling some EM structures.

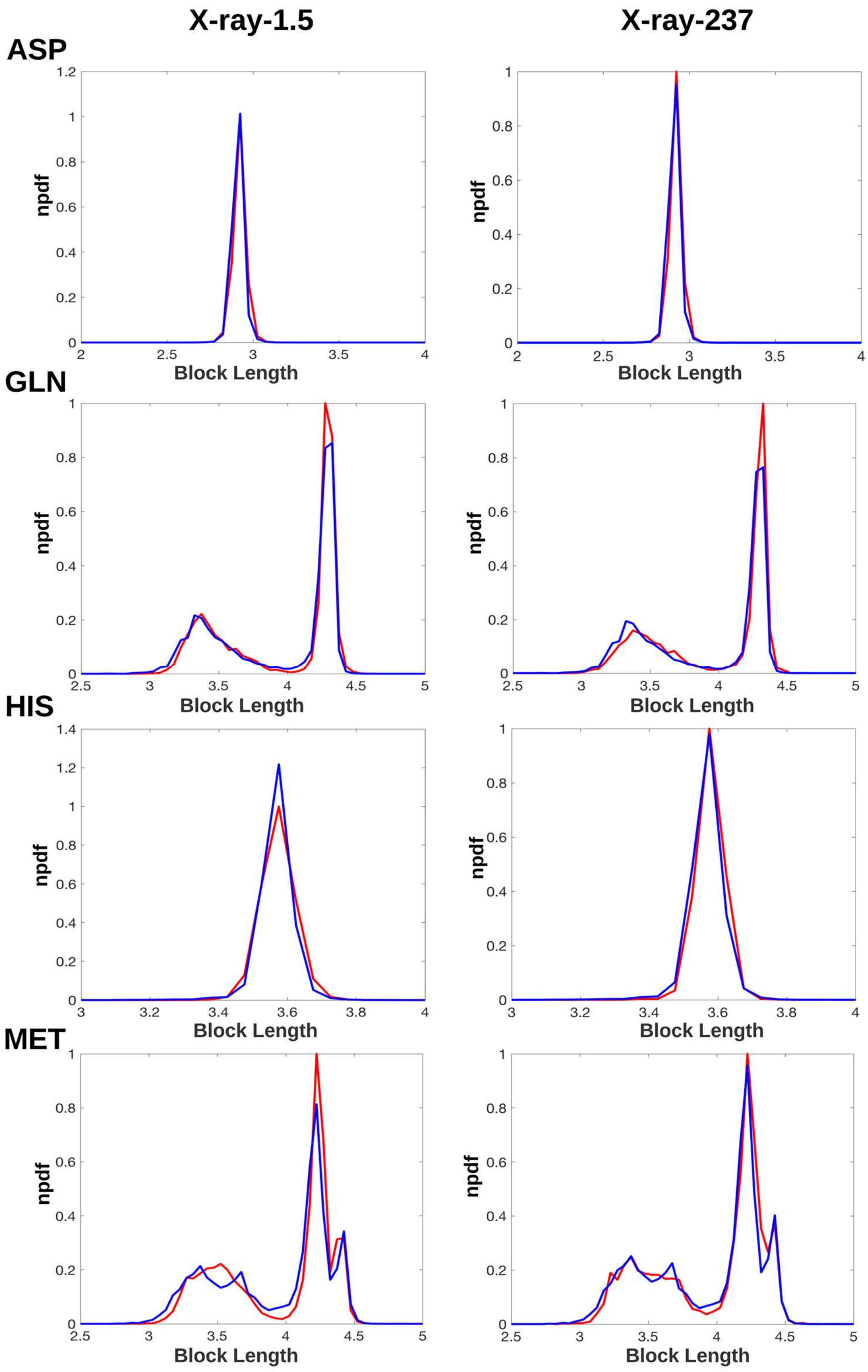

Figure S2. The npdf for Aspartic acid (ASP), Glutamine (GLN), Histidine (HIS) and Methionine (MET). The plots of left columns were generated from X-ray-1.5 (red line) and EM-2-4 (blue line); the plots of right column were generated from X-ray-237 (red line) and EM-2-4 (blue line).

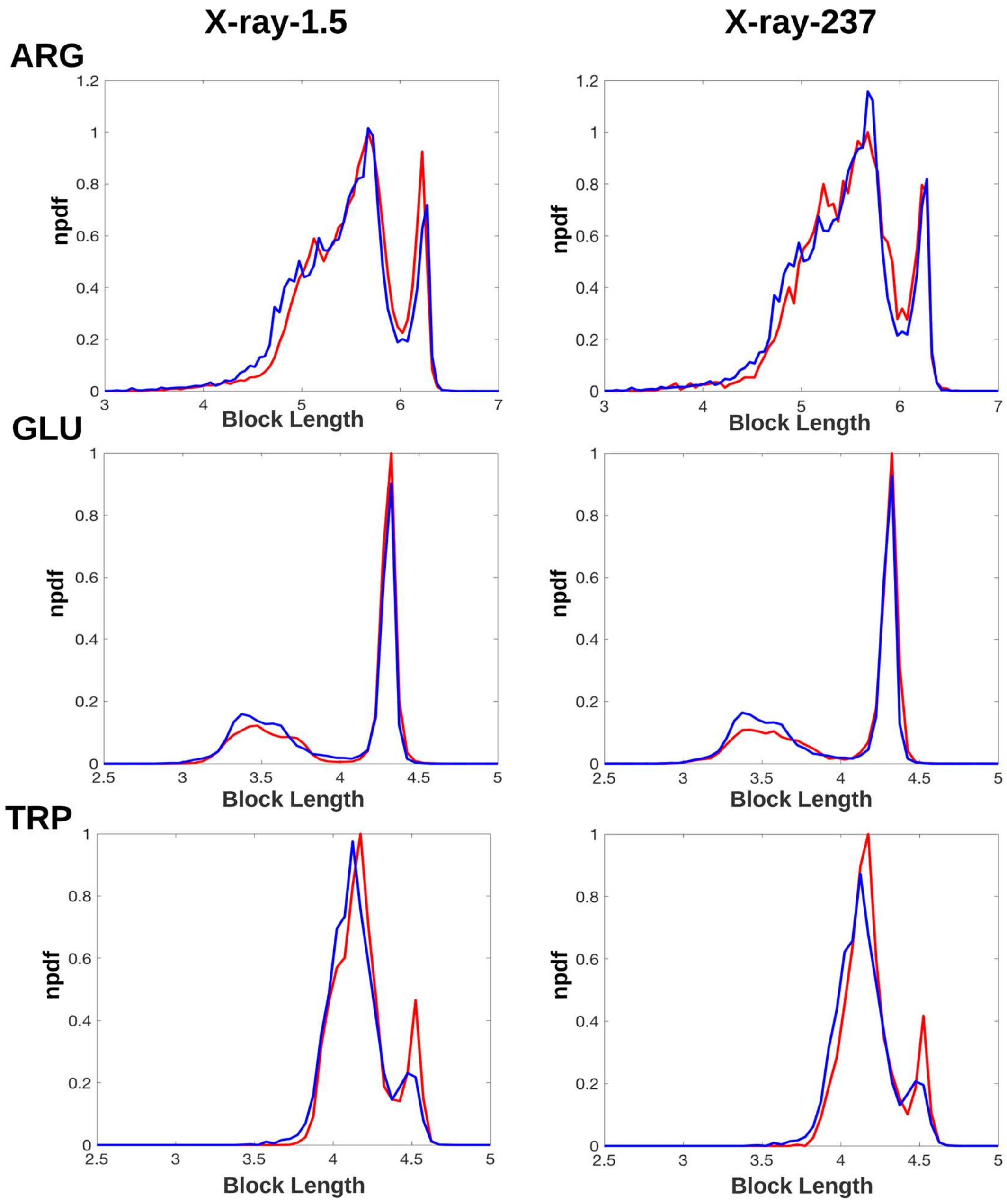

Figure S3. The npdf of block lengths for Arginine (ARG), Glutamate (GLU), Tryptophan (TRP). The plots in the left column were derived from X-ray-1.5 (red line) and EM-2-4 (blue line); the plots in the right column were derived from X-ray-237 set (red line) and EM-2-4 set (blue line).
